# Integrating soil bioavailability and plant physiological responses to establish region-specific safety thresholds for cadmium and arsenic in rice cultivated in karst regions

**DOI:** 10.3389/fpls.2025.1703651

**Published:** 2025-12-16

**Authors:** Dexin Wei, Yanlin Huang, Zhihua Yu, Linyan Zan, Weizhen Li, Kaixuan Wei, Yuhui Peng, Xueli Wang

**Affiliations:** 1 School of Agriculture, Guangxi University, Nanning, China; 2 Guangxi Key Laboratory for Agro-Environment and Agro-Products Safety, Nanning, China; 3 National Demonstration Center for Experimental Plant Science Education, Nanning, China

**Keywords:** cadmium bioavailability, arsenic bioavailability, karst region, paddy soil, high geochemical background, safe utilization, metal tolerance, sustainable agriculture

## Abstract

The national soil risk screening values (RSVs) and intervention values (RIVs) have limited applicability in karst regions with high geochemical backgrounds, often leading to unnecessary remediation efforts. To address this issue, 125 paired soil–rice samples from Gejiu–Mengzi, Yunnan Province, China, were analyzed. We found that the RSV misclassification rates (mainly false positives) for cadmium (Cd) and arsenic (As) in neutral-to-alkaline paddies (pH > 6.5) ranged from 52.0–66.4%. Soil iron/manganese (Fe/Mn) oxides and pH were identified as critical factors controlling Cd/As bioavailability in rice. This decoupling is primarily governed by soil alkalinity and abundant Fe/Mn oxides, which immobilize metals despite high total concentrations. This also explains why there is such a high rate of false positives. By integrating multiple linear regression and species sensitivity distribution (SSD) models, we derived regional safety thresholds (STs) and hazard thresholds (HTs). The newly established STs (Cd: 4.54–7.12 mg·kg^-1^; As: 91.43–92.30 mg·kg^-1^) were significantly higher than the national RSVs (Cd: 0.6–0.8 mg·kg^-1^; As: 20–25 mg·kg^-1^). The HT for Cd (9.66–10.12 mg·kg^-1^) was 2.5–3.2 times greater than the RIV for Cd, whereas the HT for As (96.90–97.15 mg·kg^-1^) was slightly lower than the RIV for As (100–120 mg·kg^-1^). Applying the ST–HT system increased the soil quality assessment accuracy from 28.1–95.6% to 71.8–100%, drastically reducing the need for unnecessary remediation. This study not only serves as a region-specific framework for safe rice cultivation in karst areas of Yunnan, but also highlights the potential for integrating soil–plant system bioavailability with physiological tolerance mechanisms. Furthermore, we provide a crucial benchmark for evaluating innovative remediation strategies aimed at alleviating metal stress in plants.

## 1 Introduction

The translocation of cadmium (Cd) and arsenic (As) from contaminated soils into food crops is a primary pathway for human exposure to these toxic elements. Their heightened risks stem from a combination of high mobility, strong bioaccumulation potential, chronic exposure, and synergistic toxicity (Wei et al., 2018; Mostofa et al., 2019; Zhong et al., 2023). Among staple foods, rice (*Oryza sativa L*.) is particularly efficient at accumulating Cd and As, posing a significant health threat, especially in Asian countries where it is a dietary cornerstone (Arunakumara et al., 2013; Luo et al., 2018). In China, rapid industrialization and mining activities have exacerbated Cd/As contamination in paddy soils (Williams et al., 2009; Kien et al., 2010; Lei et al., 2010; Krishna et al., 2013; Hang et al., 2015; Hu et al., 2016; Liu et al., 2016; Pan et al., 2016; Huang et al., 2020). In response, stringent national standards have been established, including the maximum allowable concentrations (MACs) in rice (GB 2762-2022) (National Health Commission, PRC and State Administration for Market Regulation, 2022) and the risk control standard for soil contamination of agricultural land (GB 15618-2018), which defines risk screening values (RSVs) and risk intervention values (RIVs) based primarily on soil pH (Ministry of Ecology and Environment, PRC, 2018). However, a critical limitation of these national standards is their ‘one-size-fits-all’ approach. Field evidence increasingly shows that soil–metal–rice transfer is governed by a complex interplay of local soil properties beyond just pH, often leading to significant misclassification (e.g., false positives) when national RSVs/RIVs are applied uniformly across diverse regions (Li et al., 2008; Liu et al., 2015; Zhao et al., 2015; Chen et al., 2016). Applicability in specific areas needs to be addressed urgently.

This issue of regional inapplicability is particularly acute in the karst regions of southwestern China, which are characterized by naturally high geochemical backgrounds of heavy metals due to endemic mineralization (Liu et al., 2023, 2024a; Ma et al., 2024). Yunnan Province is a quintessential example, which is a major rice-producing area overlaying vast mineral deposits, creating a stark conflict between agriculture and inherent geological enrichment (Zhao et al., 2009b; Cheng et al., 2021; Liu et al., 2024c). In such areas, simply applying national RSV/RIV can result in the widespread misclassification of safe farmland as contaminated, triggering unnecessary and costly remediation efforts. The soils in these karst areas are often neutral to alkaline and rich in iron (Fe) and manganese (Mn) oxides, which are known to be key carriers of heavy metals and play a crucial role in immobilizing Cd through adsorption/coprecipitation and regulating As bioavailability through redox processes (Fabisch et al., 2013; Zhou et al., 2015; Liu et al., 2019, 2024d; Gao and Li, 2021; Yang et al., 2021; Li et al., 2022). However, the current standards fail to account for the profound impact of these soil constituents. Consequently, there is a pressing need to develop regionalized thresholds that integrate these critical local soil parameters to achieve more accurate and economically sustainable risk assessments.

Establishing accurate regional thresholds thus requires integrating both soil bioavailability and plant physiological responses, which can vary significantly by region and cultivar. Furthermore, understanding the physiological mechanisms by which rice plants mitigate Cd and As stress, such as metal sequestration in roots (Chen et al., 2024), transporter gene regulation (Liu et al., 2024b), and antioxidant defense systems (Wang et al., 2025), can provide a biological basis for establishing increasingly accurate safety thresholds. Recent advances in biotechnology, including molecular markers and gene editing, offer promising tools for enhancing metal tolerance in rice cultivars.

Despite the recognized importance of these factors, predictive models for Cd/As transfer in soil–rice systems under real-field conditions in karst areas with high backgrounds remain scarce. Most existing models are derived from pot experiments with exogenous metal spikes, which poorly simulate complex interactions in naturally contaminated, heterogeneous field soils (Ding et al., 2013; Yang et al., 2013; Li et al., 2014; Ye et al., 2014). To bridge this gap, this study was conducted in the Gejiu–Mengzi area of Yunnan Province, which is a typical karst high-background region. We integrate field-based soil–plant transfer models with guidance from plant stress physiology to develop regionally adaptive thresholds, thereby contributing to the innovative integration of environmental (Cheng et al., 2023)and biological strategies for sustainable rice production in metal-stressed environments. We aimed to (1) evaluate the applicability and misclassification rates of the national RSV/RIV system for rice cultivation in this typical karst high-background region (such Yunnan); (2) identify the key soil factors (particularly Fe/Mn oxides and pH) controlling Cd and As bioavailability and accumulation in rice; and (3) derive scientifically definitive regional safety thresholds (STs) and hazard thresholds (HTs) for Cd and As in paddy soils by integrating multiple regression and species sensitivity distribution (SSD) models, providing a robust basis for precise risk management and safe rice cultivation.

## 2 Materials and methods

### 2.1 Study area and sampling

**Figure 1:**
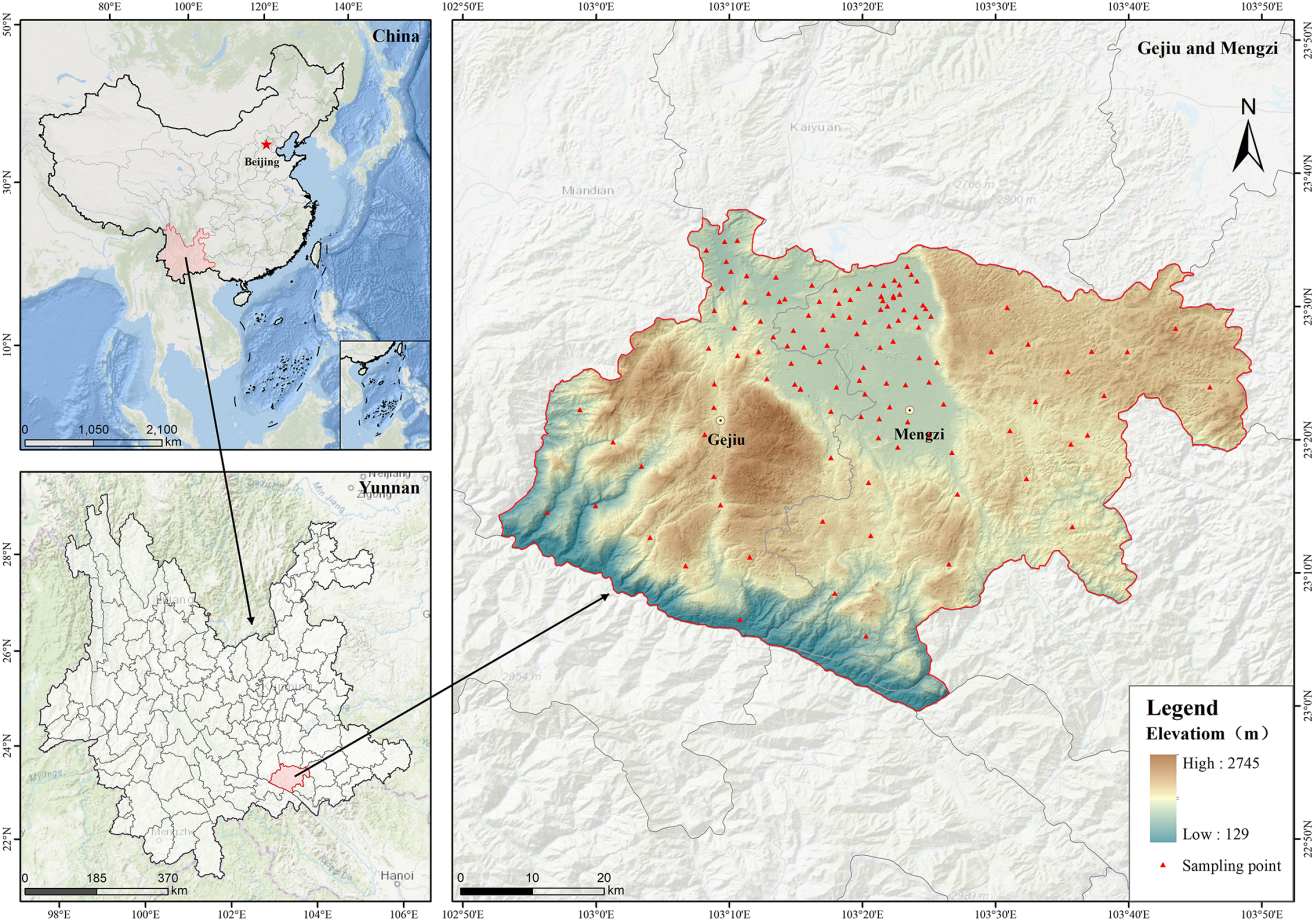
Location of the study area and spatial distribution of the 125 sampling sites.

The study area is located in Gejiu city and Mengzi city of Honghe Prefecture, both of which are located in southeastern Honghe Prefecture. This region is dominated by mountainous landforms, with fertile alluvial dam areas distributed among them, and sufficient solar and thermal resources provide superior conditions for agricultural production (**Figure 1**). Characterized by a typical low-latitude plateau subtropical monsoon climate, the area has a mean annual temperature of 18.6 °C. This region is a typical karst region.

On the basis of the spatial distribution map of the mining area in Yunnan Province, the planting zoning map of Honghe Prefecture, and the investigation of heavy metal pollution in crops and soil, a stratified random sampling design was implemented: grids at 1×1 km intervals covered the northern contiguous rice farming area, while the scattered farmland in the southern area allowed the sampling grid to be appropriately expanded to 3 × 3 km. Rice–soil collaborative sampling was conducted in the rice-growing area of the study area from September 3 to 28, 2018. By using stainless steel soil drills (Ø 5 cm), soil samples were collected from the 0–20 cm layer of the cultivated soil. The center position of the sampling unit was determined using GPS. The samples were sealed in sterile EO-sterilized polyethylene sampling bags after plant residues, gravel, and weeds were removed. Three to five mature rice plants within a 5-meter range were simultaneously collected using ceramic scissors. The GPS coordinates, collection time, sample ID, and metadata were all fully recorded. A total of 125 paired soil–rice samples were obtained.

### 2.2 Sample pretreatment and chemical analysis

All the soil samples were air-dried, ground, and sieved prior to chemical analysis. The TRice grains were oven-dried, dehusked to produce milled rice, pulverized, and sieved through a 0.149-mm (100-mesh) sieve. Soil pH was measured using a glass electrode with a 1:2.5 soil:water (w/w) suspension. The soil organic matter (SOM) content was quantified via the external heating method using concentrated H_2_SO_4_ and K_2_Cr_2_O_7_. Total Cd/As digestion employed a mixed-acid solution (HCl: HNO_3_:HClO_4_ = 1:1:1, v/v/v) followed by atomic fluorescence spectrometry (PinAAcle900T, PerkinElmer Instruments Co., Ltd). Cd/As concentrations in soil and rice were analyzed using HNO_3_+HClO_4_ (4:1 v/v; GR grade) digestion coupled with inductively coupled plasma–mass spectrometry (Nexion 350x, PerkinElmer Instruments Co., Ltd). Iron and manganese oxides were quantified via flame atomic absorption spectrophotometry (iCE 3000 Series, Thermo Fisher Scientific Inc.), for specific methods, please refer to the **Supplementary Methods**. Quality control measures: During the testing process, for every 10 samples, 1 soil standard sample (GBW-07405) or 1 plant standard sample (GBW-10045) and 1 blank sample were inserted, and the recovery rates of the added substances were determined.

### 2.3 Regression model based on field survey data

Multivariate regression analysis was conducted on soil pollutant content (C*_Soil_*, mg·kg^-1^), rice pollutant content (C*_Rice_*, mg·kg^-1^), and soil physicochemical properties (such as pH) on the basis of field collaborative (point-to-point sampling) survey data of soil and agricultural products. In addition, the prediction model was constructed. In order to ensure that the data met the normal distribution and reduce the difference in the order of magnitude of the data, we performed a base 10 log transformation on the pair of data. The threshold of heavy metals in soil was derived. The regression equation is shown in Equation 1:

(1) $$\mathrm{lg}\left(C_{Rice}\right)=a\times \mathrm{lg}\left(C_{Soil}\right)+b\times pH+c$$

In the formula, *C_Rice_* is the pollutant content in rice (mg·kg^-1^), *C_Soi_*_l_ is the content of pollutants in soil (mg·kg^-1^), pH is the soil pH value (dimensionless), and a, b, and c are the parameters.

### 2.4 SSD based on Daejeon survey data

The sensitivity distribution of the soil–crop pollutant enrichment effect was obtained through field survey data, and the soil threshold could be derived according to the protection of different proportions of rice varieties. The enrichment effect of rice on soil pollutants could be described by the bioconcentration factor (*BCF*, %), which is the ratio of *C_Rice_* to *C_Soil_*, as shown in Equation 2.

(2) $$BCF=\frac{C_{Rice}}{C_{Soil}}\times 100\%$$

The sensitive distribution of crops collected in field investigations to the concentration effect of pollutants in soil should follow the “S” curve distribution, and the crop enrichment factor and accumulation probability were fitted using a logistic model. Equation 3 is expressed as follows:

(3) $$y=\frac{a}{1+(\frac{x}{x_{0}}{)}^{b}}$$

where x is 1/BCF, y is the cumulative probability (%) of the crop sample corresponding to the value of x, and a, b, and x_0_ are constants.

The value of 1/BCF with the risk of exceeding the standard for different proportions of rice was reversely deduced by Equation 3, as shown in Equation 4. Taking Cd as an example, according to MAC-Cd=0.2 mg·kg^-1^, the Cd threshold in soil (*C_soil_*) was derived according to Equation 5:

(4) $$\frac{1}{BCF}=10^{\frac{lg(\frac{a}{y}-1)}{b}}+lgx_{0}$$

(5) $$C_{Soil}=\frac{1}{BCF}\times C_{Rice}$$

### 2.5 Data analysis

The experimental data were processed by Excel 365, and normality, correlation and multiple regression analyses were performed with SPSS 27.0. All the samples (n=125) were randomly divided into modeling samples (n=116) and validation samples (n=9). To ensure that the data met the normal distribution and reduce the number difference of the data, logarithmic transformation with a base of 10 was conducted. The modeling samples were used to construct a prediction model for rice Cd and As contents, and the verification samples were used to check the accuracy of the model. With Origin 2021, GraphPad Prism (version 9.5), Arcgis (version 10.8) and EEC–SSD mapping.

## 3 Results

### 3.1 Basic properties of soil and contents of Cd and As in rice

**Figure 2:**
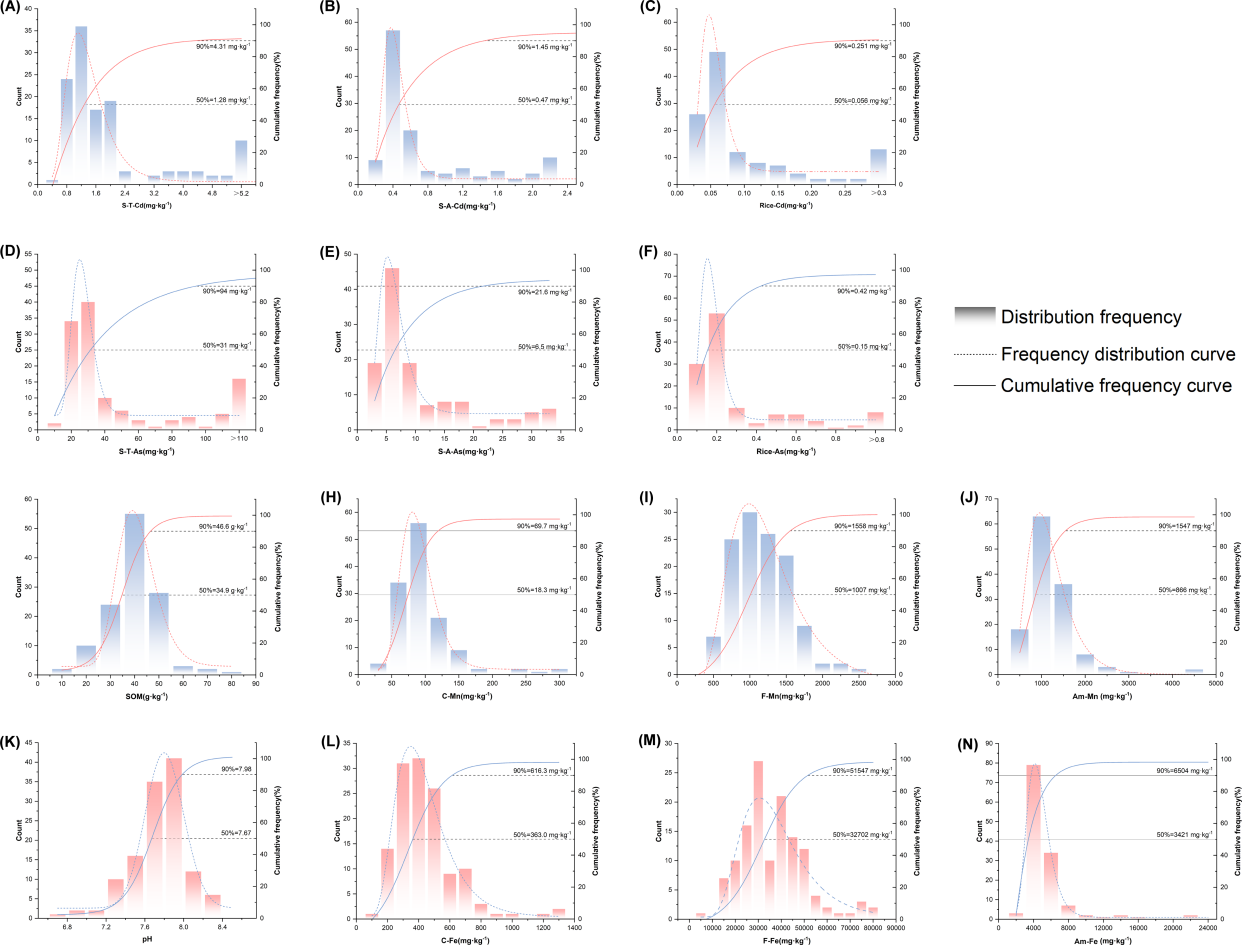
Frequency distribution histograms of soil properties and Cd/As concentrations in soil and rice. **(A)** Soil total–Cd(S-T-Cd), **(B)** available–Cd(A-Cd), **(C)** rice–Cd(Rice-Cd), **(D)** soil total–As(S-T-As), **(F)** rice–As(Rice-As), **(G)** SOM, **(H)** complex–Fe(C-Fe), **(I)** free–Fe(F-Fe), **(J)** amorphous–Fe(Am-Fe), **(K)** pH, **(L)** complex–Mn(C-Mn), **(M)** free–Mn(F-Mn), and **(N)** amorphous–Mn(Am-Mn).

The pH values of the paddy soil in the karst high geological back area of Yunnan are neutral or alkaline, and the enrichment characteristics of Cd and As are significant. The soil samples exhibit broad physicochemical variability (**Supplementary Table S1**; **Figure 2**). Given that the study area is in a karst region, the contents of Cd and As in the soil are relatively high, with average values of 2.16 and 60.18 mg·kg^-1^, respectively. The soil pH ranges from neutral to weakly alkaline (6.56–8.25; mean = 7.65). Weakly alkaline soils (7.5< pH ≤ 8.5) account for 74.4% of the samples, whereas neutral soils (6.5< pH ≤ 7.5) account for 25.6%. Soil alkalization can lead to essential micronutrient deficiencies, posing a major challenge to rice cultivation. Conversely, the soil organic matter (SOM) content ranges from 9.53 to 70.30 g·kg^-1^ (mean = 34.55 g·kg^-1^), which is indicative of the SOM content in the study area. This indicates that our data can effectively reflect the soil characteristics within the study area.

### 3.2 Evaluation of the suitability of paddy field soil quality by the RSV

**Figure 3:**
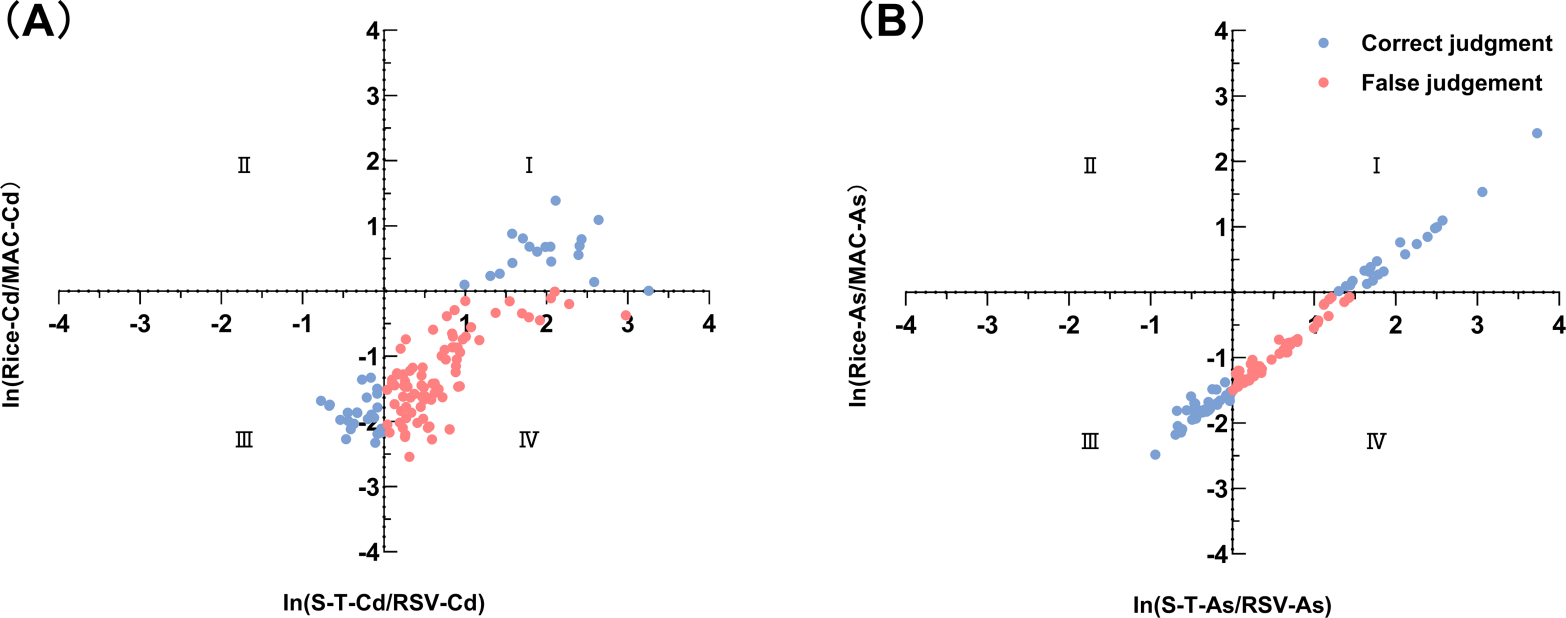
Evaluation of the applicability of the national risk screening value (RSV) suitability evaluation: **(A)** Cd and **(B)** As. The coordinate axes represent the ln-transformed thresholds of the RSV (vertical) and the MAC (horizontal), dividing the plot into four quadrants: I (true positive), II (false negative), III (true negative), and IV (false positive). The high density of points in Quadrant IV indicates a severe overestimation of risk by the national standard.

The current national standard (RSV) is seriously inadequate in terms of its applicability to neutral-to-alkaline paddy fields, and it has a pH greater than 6.5. The measured amounts of S–T–Cd and S–T–As in each soil sample are compared with those of RSV–Cd and RSV–As, respectively, to assess whether the soil can be safely used for rice cultivation. Soils are classified as safe (S–T–Cd< RSV–Cd or S–T–As< RSV–As) or unsafe (S–T–Cd > RSV–Cd or S–T–As > RSV–As). Paired rice Cd/As concentrations are then compared with maximum allowable concentrations (MACs) to assess the applicability of the RSV. If the contents of Cd and As in soil and milled rice in the same plot are higher or lower than the corresponding standard, the RSV is considered suitable. A four-quadrant plot is drawn to illustrate the evaluation results (**Figure 3**).

Quadrant (I): ln (S–T–Cd/RSV–Cd) > 0 and ln (rice–Cd/MAC–Cd) > 0, or ln (S–T–As/RSV–As) > 0 and ln (rice–As/MAC–As) > 0, soil unsafe, rice unsafe, and RSV suitable.

This quadrant contains 18 Cd-exceeding samples, with a mean soil Cd concentration of 6.14 mg·kg^-1^ and a mean rice Cd concentration of 0.381 mg·kg^-1^. For As, the mean levels are 207.76 mg·kg^-1^ (soil) and 1.12 mg·kg^-1^ (rice). Thus, 14.4–17.6% of soils are unsafe for rice production, enabling the entry of Cd/As into the food chain. Consequently, remediation is needed to minimize Cd transfer and ensure food safety.

Quadrant (ii): ln(S–T–Cd/RSV–Cd)< 0 and ln(rice–Cd/MAC–Cd) > 0 or ln(S–T–As/RSV–As)< 0 and ln(rice–As/MAC–As) > 0; soil safety, rice insecurity, and RSV unsuitable (false negative).

This quadrant indicates that the soil sample is assessed as safe for rice cultivation; however, the Cd or As content of the produced rice exceeds the limit requirements for MAC, the rice is unsafe, and the assessment results are false negatives. However, there are 0 paired samples in this quadrant, indicating that RSV greatly protects rice planted in the study area.

Quadrant (III): ln(S–T–Cd/RSV–Cd)< 0 and ln(rice–Cd/MAC–Cd)< 0 or ln(S–T–As/RSV–As)< 0 and ln(rice–As/MAC–As)< 0, soil safety, rice safety, and RSV suitability.

This quadrant indicates that the soil sample is assessed as safe for rice cultivation; moreover, the Cd or As content of the produced rice does not exceed the limit requirements for MAC, and the rice is safe. In this quadrant, there are 24 Cd-based safe paired samples and 38 As-based safe paired samples.

Quadrant (IV): ln(S–T–Cd/RSV–Cd) > 0 and ln(rice–Cd/MAC–Cd)< 0 or ln(S–T–As/RSV–As) > 0 and ln(rice–As/MAC–As)< 0; the soil is unsafe, the rice is safe, the RSV is unsuitable, and false positives are present.

This quadrant indicates that the soil sample is incorrectly assessed as unsafe for rice cultivation; however, the Cd or As content in the produced rice does not exceed the limit requirements of the MAC, indicating false positive evaluation results. There are 83 false-positive paired samples of Cd and 65 false-positive paired samples of As in this quadrant, and the misdiagnosis rate ranges from 52.0% to 66.4%. Therefore, the RSV evaluation criteria are overly strict and that the RSV may have overestimated the risks associated with rice production in the study area.

### 3.3 Soil controlling factors affecting the contents of Cd and As in rice

**Figure 4:**
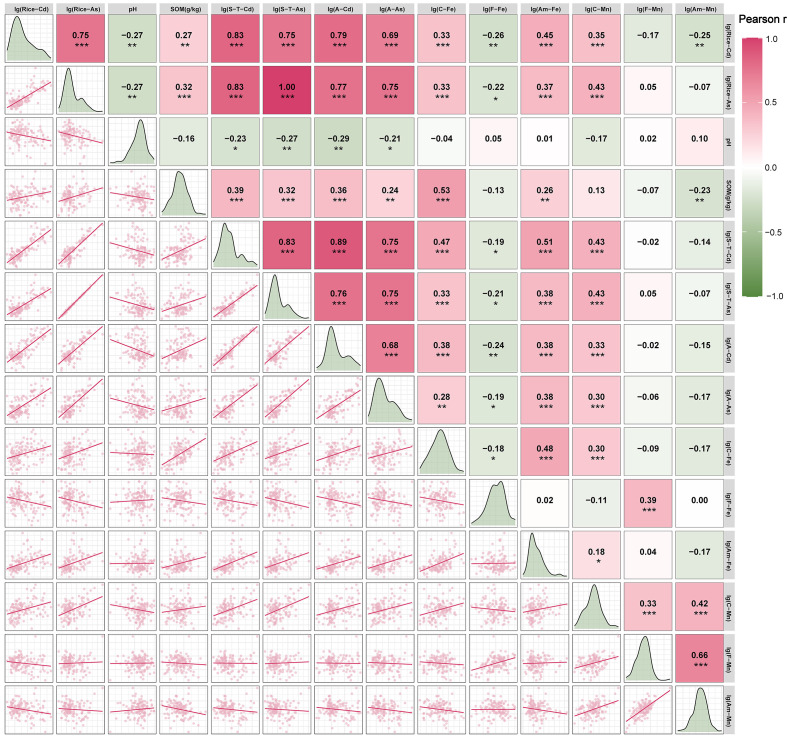
Pearson correlation heatmaps of Cd/As concentrations in rice and soil properties. * indicates *P<* 0.05; ** indicates *P* < 0.01; *** indicates *P* < 0.001. The color intensity and value in each cell represent the correlation coefficient. S–T, soil total; A, available; C, complex; F, free; Am, amorphous.

SOM, pH and Fe/Mn oxides regulate the enrichment of Cd and As in rice in high karst geological areas. To explain safe rice production under high soil Cd/As loads in karst regions, we analyze correlations between soil properties and rice Cd/As concentrations. To satisfy the normality of the data and reduce the quantitative difference between them, logarithmic transformation is performed on all the data except for the pH and SOM. The correlation results (**Figure 4**) reveal that rice–Cd is positively correlated with S–T–Cd, A–Cd, C–Fe, A–Fe and C–Mn (P<0.001); positively correlated with SOM (P<0.01); and negatively correlated with pH, F–Fe and A–Mn (P<0.01). Rice–As is positively correlated with SOM, S–T–As, A–As, C–Fe, A–Fe, and C–Mn (P<0.001), negatively correlated with pH (P<0.01), and negatively correlated with F–Fe (P<0.05). Thus, these factors primarily govern Cd/As accumulation in rice.

### 3.4 Prediction model for Cd and As contents in rice

#### 3.4.1 Prediction model for Cd and As contents in rice

Our correlation analysis (**Figure 4**) reveals the differential effects of soil factors on rice Cd/As accumulation. The significantly correlated soil factors are selected as independent variables, with rice Cd/As serving as the dependent variable. Among the 125 paired samples, 116 are allocated as training sets for multiple linear regression. As seen from the correlation analysis results of the Cd and As contents in rice and various soil factors (**Figure 4**), various soil factors affect the accumulation of Cd and As in rice. Soil factors significantly that are correlated with the Cd and As contents in rice are screened, and the contents of Cd and As in rice are considered as the dependent variables. Soil factors that are significantly correlated with the contents of Cd and As in rice are used as independent variables, and 116 pairs of paired samples are separated from 125 pairs as training sets for linear multiple regression analysis. Regression analysis indicates that there is severe collinearity between S–T–Cd and A–Cd (VIF > 5). Given their high correlation (**Figure 4**, P = 0.89 > 0.7), A–Cd is excluded. The final rice Cd/As prediction models are presented in **Table 1**.

**Table 1:** Prediction model of Cd and As contents in rice.

Model	Regression equation	R	R^2^	Adjusted R^2^	*P*
Model(S-T-Cd)	lg(Rice-Cd)=1.481-0.096×pH-0.003×SOM+0.852×lg(S-T-Cd)-0.25× lg(C-Fe)-0.32×lg(F-Fe)+0.217×lg(Am-Fe)+0.238×lg(C-Mn)-0.381×lg(Am-Mn)	0.861	0.742	0.724	<0.001
Model(S-T-As)	lg(Rice-As)=-2.077-0.006×pH-0.000125×SOM+0.975×lg(S-T-As)-0.003×lg(A-As) -0.001×lg(C-Fe)-0.017×lg(F-Fe)-0.006×lg(Am-Fe)-0.002×lg(C-Mn)	0.999	0.998	0.997	<0.001

Modeling sample n=116. S-T, soil total; A, available; C, complex; F, free; Am, amorphous.

The degree of fit of the equation model is expressed by the adjusted R^2^, and the closer the adjusted R^2^ is to 1, the better the degree of fit of the equation. The correlation coefficient R of model(S–T–Cd) is 0.861, which is greater than 0.7, indicating that there is a high correlation between the independent and dependent variables. The adjusted R^2^ is 0.724, indicating that 72.4% of the variation in the dependent variable lg(rice–Cd) is caused by the independent variable X; that is, pH, SOM, S–T–Cd, C–Fe, F–Fe, A–Fe, C–Mn, and A–Mn significantly explain 72.4% of the variation in rice–Cd. The correlation coefficient R of model(S–T–As) is 0.999, and the adjusted R^2^ is 0.997, indicating that 99.7% of the variation in the dependent variable lg(rice–Cd) is caused by the independent variable X. Specifically, pH, SOM, S–T–As, A–As, C–Fe, F–Fe, A–Fe, and C–Mn explain 99.7% of the variation in rice–Cd, and the result is significant (*P<* 0.001).

#### 3.4.2 Relative importance of the model parameters

The multiple linear regression model can predict not only the contents of Cd and As in rice but also the degree of contribution of each soil factor to the contents of Cd and As in rice, which is helpful for further explaining the dependence between Cd and As in rice and each soil factor.

**Figure 5:**
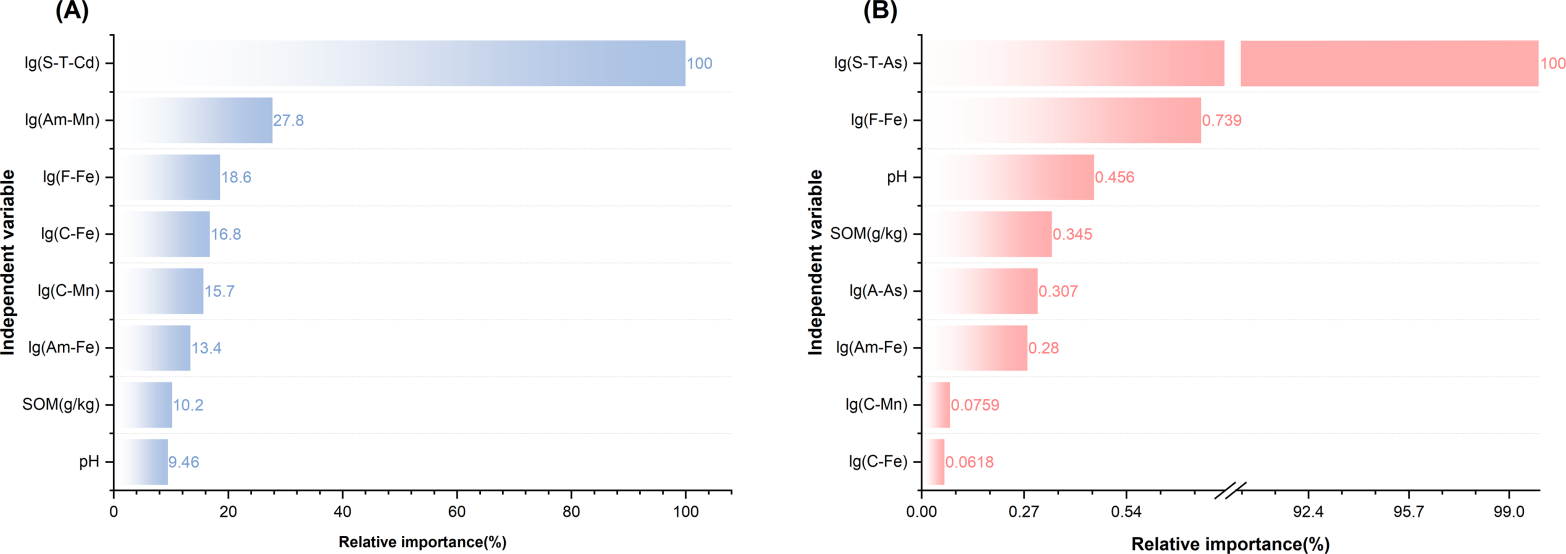
Relative importance of predictor variables in the multiple linear regression models for **(A)** rice Cd and **(B)** rice As. S-T, soil total; A, available; C, complex; F, free; Am, amorphous.

The relative variable importance for both models is shown in **Figure 5**. S–T–Cd and S–T–As have the greatest influence (100% normalized importance), followed by A–Mn (27.8%) and F–Fe (0.739%). Notably, in model(S–T–As), the difference between S–T–As and F–Fe, the relative importance of S-T-As is 135.3 times that of the second-ranked F-Fe., indicating that S–T–As is dominant in model(S–T–As) and that S–T–As is the most important factor affecting the absorption of As by rice.

#### 3.4.3 Model verification

**Figure 6:**
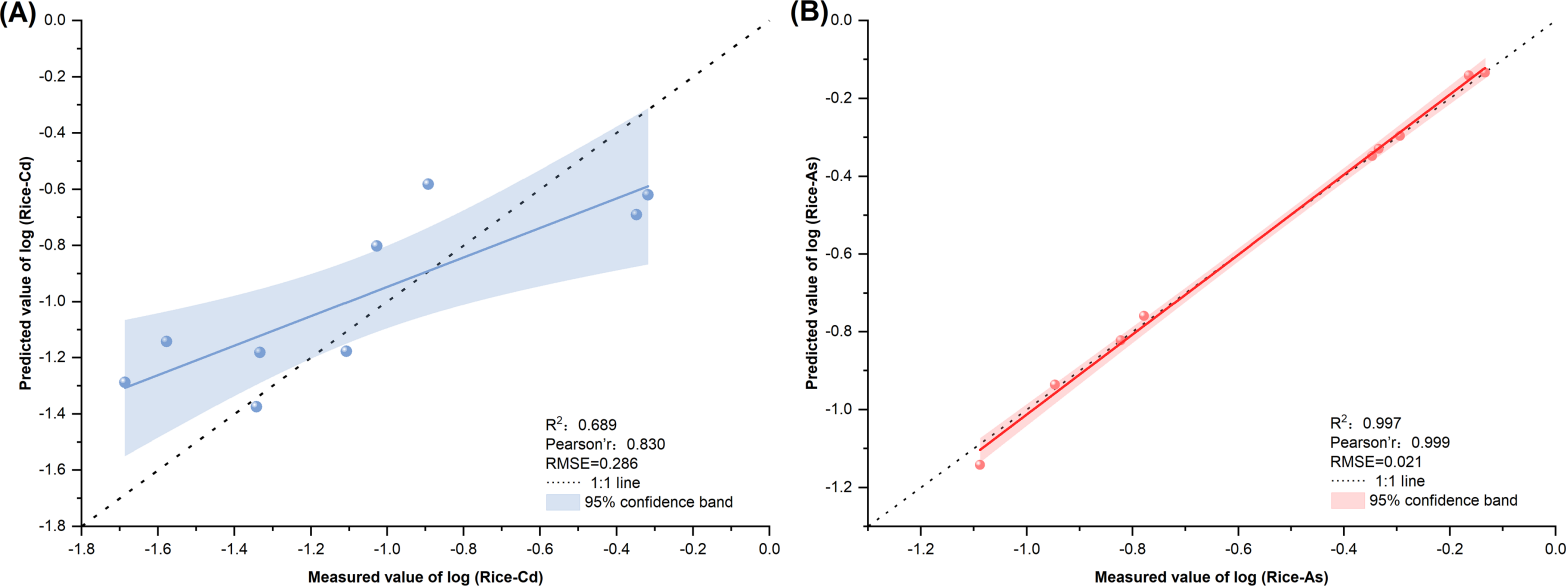
Comparison of the predicted and measured values of Cd and arsenic in rice by different regression models: **(A)** comparison between the predicted value of model(S–T–Cd) and the measured value and **(B)** comparison between the predicted value of model(S–T–As) and the measured value.

A validation set sample (n=9) is used to test the model. Through fitting analysis, we compare predicted and measured rice Cd/As to evaluate the accuracy. A higher R^2^ (closer to 1) and lower RMSE indicate a better fit. The results in **Figure 6** show that the R^2^ of each fitting equation is above 0.5, and the correlation between the predicted value and the measured value is good.

For model(S–T–Cd), R^2^ = 0.689 and RMSE = 0.286; these predictions are aligned with the 1:1 line within the 95% CI (**Figure 6A**). Model(S–T–As) achieves an R^2^ = 0.997 and an RMSE = 0.021 (**Figure 6B**). Most predictions fall within the 95% CI and are clustered along the 1:1 line, confirming excellent accuracy.

### 3.5 Determination of the soil heavy metal threshold

#### 3.5.1 Basic rules for determining thresholds

The established thresholds of Cd and As in paddy soil are divided into safety thresholds (STs) and hazard thresholds (HT)s. The ST represents the critical contents of Cd and As in soil when the corresponding contents in rice exceed the standard levels. HT indicates soil concentrations damaging 95% of the rice plants. ST and HT correspond to RSV and RIV, respectively. The method of determining the threshold refers to the formulation method titled “Soil Pollution Risk Control Standard Agricultural Land Soil Pollution Risk Screening Value and Control Value (Trial)”.

The Cd safety threshold (ST–Cd) and As safety threshold (ST–As) of paddy soil in the Gejiu–Mengzi area of Yunnan Province are deduced on the basis of the maximum allowable contents of Cd and As in the MAC. In accordance with the current soil environmental quality standards, the soil pH in this study is divided into two sections: 6.5< pH ≤ 7.5 (pH=7) and > 7.5 (pH=8). The average values of SOM, soil iron oxide and manganese oxide are taken from all the sampling points in the study area. The SOM concentration is 34.55 g·kg^-1^, the C–Fe concentration is 390 mg·kg^-1^, the C–Mn concentration is 80 mg·kg^-1^, the F–Fe concentration is 34,450 mg·kg^-1^, the F–Mn concentration is 1,080 mg·kg^-1^, the A–Fe concentration is 4,190 mg·kg^-1^, and the A–Mn concentration is 970 mg·kg^-1^. The heavy metal content prediction model of rice is assigned, The mean values of these attributes were substituted into the linear regression equation, and the contents of Cd and As in soil when Cd and As in rice reached MACs were reverse-derived using the national standard MACs as the limiting value, which were ST-Cd and ST-As.

The cumulative distribution frequency of the 95% bioenrichment coefficient of Cd and As in rice is calculated using SSD, and the HC_95_ values of Cd and As in soil are calculated on the basis of food safety standards, which are the soil thresholds for 95% of rice to be harmed by Cd and As, namely, HT–Cd and HT–As.

#### 3.5.2 Safety threshold

There is a significant positive correlation between the S-T-AS and the A-As (**Figure 3**, P = 0.75***). By establishing the regression equation for S–T–As (x) and A–As (y) (y=0.721x-0.263, R^2^ = 0.55, *P* < 0.05) and considering the linear relationship between S–T–As and A–As, we use S–T–As instead of A–As (That is, A-As=0.721*S-T-As-0.263). In accordance with model(S–T–Cd) and model(S–T–As), the ST–Cd and ST–As of paddy soil are obtained, as shown in **Table 2**. When 6.5< pH< 7.5, the ST–Cd concentration is 4.54 mg·kg^-1^, and the ST–As concentration is 91.43 mg·kg^-1^; when the pH is > 7.5, the ST–Cd concentration is 7.12 mg·kg^-1^ and the ST–As concentration is 92.30 mg·kg^-1^. The contents of ST–Cd and ST–As are greater than those of RSV–Cd and RSV–As in paddy fields, and the effects of RSV are stronger for neutral and alkaline paddy soils in karst regions.

**Table 2:** Safety threshold and risk screening value in paddy soil.

Element	pH	ST(mg·kg^-1^)	RSV(mg·kg^-1^)
Cd	6.5<pH ≤ 7.5	4.54	0.6
	pH>7.5	7.12	0.8
As	6.5<pH ≤ 7.5	91.43	25
	pH>7.5	92.30	20

#### 3.5.3 Hazard threshold

**Figure 7:**
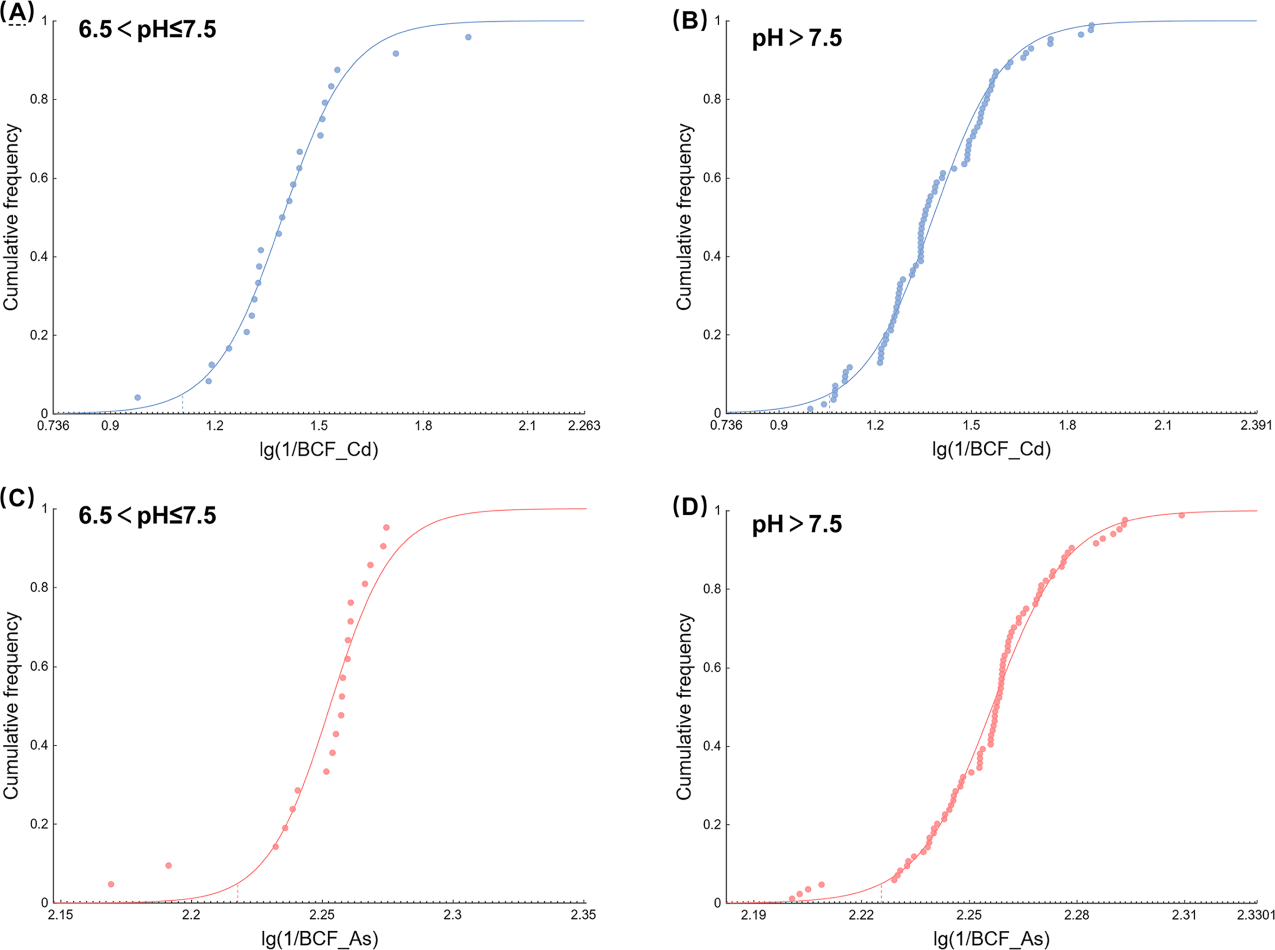
SSD curves illustrating the enrichment coefficients of Cd and As in rice at varying pH levels. **(A)** SSD curve of Cd in rice with 6.5<pH ≤ 7.5; **(B)** SSD curve of Cd in rice with pH > 7.5; **(C)** SSD curve of As in rice with 6.5< pH ≤ 7.5; and **(D)** SSD curve of As in rice with pH > 7.5.

In accordance with the soil environmental quality standards, all the sample points in the study area are divided into 2 pH intervals, and HT–Cd and HT–As are established at different pH intervals. The BCFs of Cd and As are reciprocal. The cumulative frequencies of rice Cd and As 95% are calculated by the logistic growth model of EEC–SSD software. The HC_95_–Cd and HC_95_–As value of the rice soil are calculated by combining MAC–Cd and MAC–As in the food pollutant limit; the values are called HT–Cd and HT–As, respectively. The logistic growth models fit the SSD curves well across the different pH ranges (**Figure 7**).

HT–Cd and HT–As can be obtained by using SSD, as shown in **Table 3**. When the pH is< 6.5 or ≤7.5, the reciprocal value of the rice Cd bioenrichment factor (1/BCF_Cd) corresponding to the 95% cumulative distribution frequency is 48.29, and the soil HT–Cd concentration can be estimated to be 9.66 mg·kg^-1^ with a MAC–Cd concentration of 0.2 mg·kg^-1^. When the pH is > 7.5, 1/BCF_Cd is 50.6, and the soil HT–Cd concentration is 10.12 mg·kg^-1^. The concentration of HT–Cd is greater than that of RIV–Cd, and RIV–Cd is more sensitive to neutral and alkaline paddy soil in karst regions.

**Table 3:** Hazard threshold and risk intervention value of paddy soil.

Element	pH	HT(mg·kg^-1^)	RIV(mg·kg^-1^)
Cd	6.5<pH ≤ 7.5	9.66	3.0
	pH>7.5	10.12	4.0
As	6.5<pH ≤ 7.5	97.15	120
	pH>7.5	96.9	100

When 6.5<pH ≤ 7.5, the reciprocal value of the rice As bioenrichment factor (1/BCF_As) corresponding to the 95% cumulative distribution frequency is 194.3. On the basis of MAC–As= 0.5 mg·kg^-1^, the soil HT–As concentration can be estimated to be 97.15 mg·kg^-1^. When the pH is > 7.5, 1/BCF_As is 193.8, and the soil HT-As concentration is 96.9 mg·kg^-1^. The soil HT–As predicted in this study is lower than the RIV–As but similar to the RIV–As (100 mg·kg^-1^) when the soil environmental quality standard is more than 7.5. The RIV–As is relatively relaxed for alkaline paddy soil in the karst region.

### 3.6 Application and evaluation of the ST-HT soil quality evaluation system

#### 3.6.1 Accuracy evaluation of the soil quality evaluation system based on the ST-HT

The ST–HT system has improved the accuracy of soil quality assessment. The newly established ST and HT values are used to evaluate the exceedance of soil Cd and As in the paddy field in the study area. The results are compared with those of the RSV–RIV benchmarks. **Table 4** shows low accuracy for the GB 15618–2018 RSV–RIV system. The use of the ST–HT soil environmental assessment system improves the accuracy of heavy metal concentrations to exceed the standard in the soil–rice system, especially in the prediction of As. The accuracy increases from 43.0–100% to 96.9–100% under ST–HT.

**Table 4:** The accuracy of different evaluation criteria to determine the exceedance of Cd and As in soil rice.

Element	pH	Evaluation criteria	Value (mg·kg^-1^)	Accuracy (%)	False positive rate (%)	False negative rate (%)
Cd	6.5<pH ≤ 7.5	RSV	0.6	28.1	71.9	0
		ST	4.54	71.8	9.4	18.8
		RIV	3.0	75	15.6	9.4
		HT	9.66	71.9	3.1	25.0
	pH>7.5	RSV	0.8	35.5	64.5	0
		ST	7.12	95.7	0	4.3
		RIV	4.0	95.6	2.2	2.2
		HT	10.12	92.5	0	7.5
As	6.5<pH 527.5	RSV	25	68.75	31.25	0
		ST	91.43	100	0	0
		RIV	120	84.4	0	15.6
		HT	97.15	96.9	0	3.1
	pH>7.5	RSV	20	43.0	57.0	0
		ST	92.30	100	0	0
		RIV	100	100	0	0
		HT	96.9	100	0	0

#### 3.6.2 Evaluation of regionalization based on the ST–HT soil quality evaluation system

**Figure 8:**
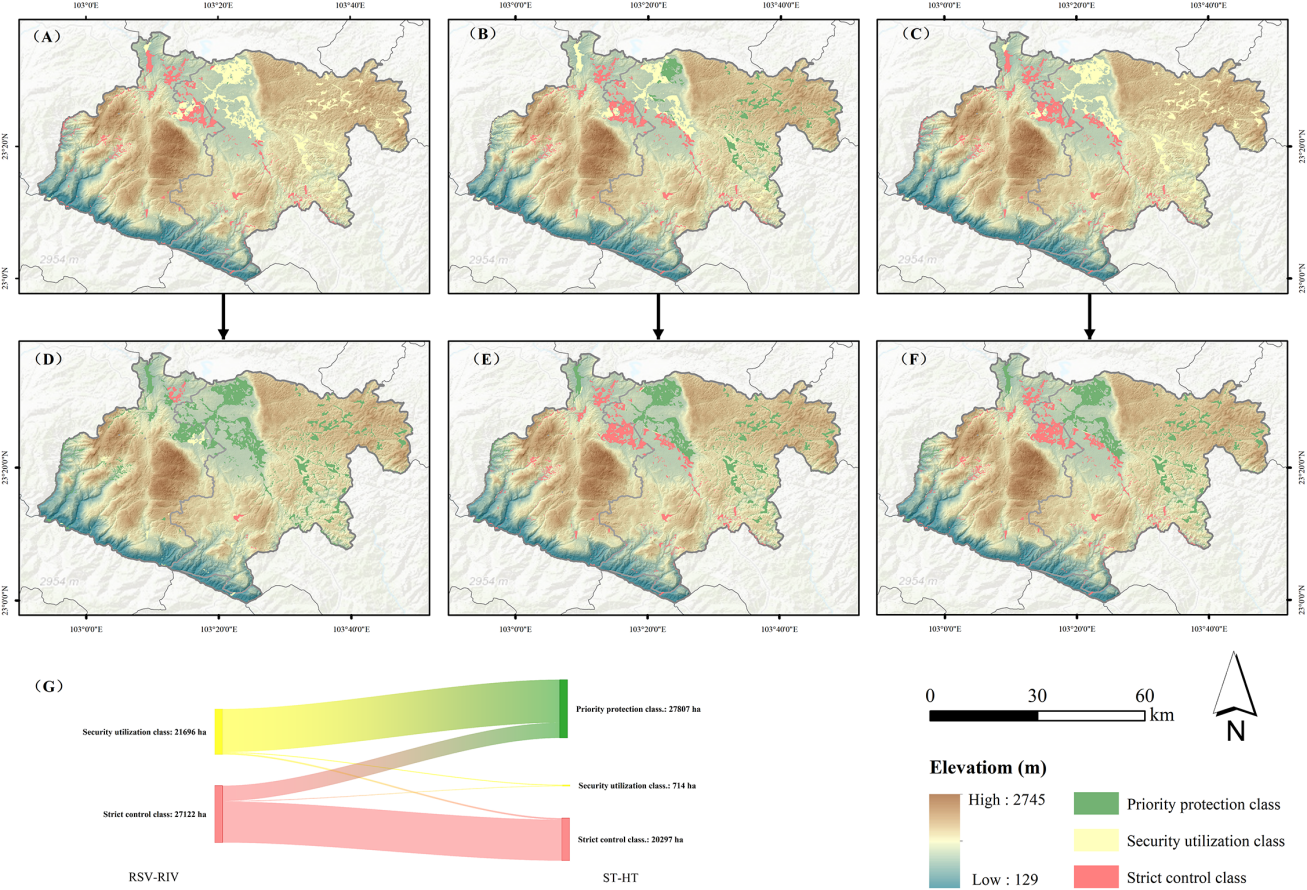
Spatial distribution and areal change of paddy soil environmental quality classes based on different evaluation systems. **(A)** Environmental quality classification of Cd pollution based on the RSV–RIV soil environmental assessment system; **(B)** As pollution environmental quality classification based on the RSV–RIV soil environmental assessment system; **(C)** comprehensive pollution environmental quality classification based on the RSV–RIV soil environmental assessment system; **(D)** environmental quality classification of Cd pollution based on the ST–HT soil environmental assessment system; **(E)** As pollution environmental quality classification based on the ST–HT soil environmental assessment system; **(F)** comprehensive pollution environmental quality classification based on the ST–HT soil environmental assessment system; and **(G)** changes in soil quality classification under the RSV–RIV and ST–HT evaluation systems.

The planting area division based on the ST–HT system significantly reduces the redundant restoration costs. The ST–HT system is applied to the safe planting zoning of rice fields in the study area. When C_i_ ≤ ST or RSV for Cd/As, the soils are unpolluted. For RSV< C_i_ ≤ RIV or ST< C_i_ ≤ HT, pollution requires enhanced crop monitoring. C_i_ > RIV or C_i_ > HT indicates that the soil is heavily contaminated by the heavy metal. Compared with RSV–RIV and ST–HT, the two evaluation systems are classified into the following types of soil environmental quality: (I) class I, C_i_< RSV or ST, low risk of soil pollution, and priority protection class and (II) class II, RSV< C_i_≤RIV or ST< C_i_≤HT, which may indicate a controllable soil pollution risk. Combined with the results of the collaborative monitoring of agricultural products, the results are divided into the following classes: (III) class III, C_i_ > RIV or C_i_ > HT, with high soil pollution risk, is classified into the strict control class on the basis of the results of the collaborative monitoring of agricultural products. Comprehensive Cd/As pollution levels are determined for the worst-case contaminants. Spatial interpolation is performed in ArcGIS 10.8 to generate soil quality maps (**Figure 8**).

Under RSV–RIV, security utilization equates to 21,696 ha, strict control equates to 27,122 ha, and there is no priority protection (**Figures 8A–C**). ST–HT yields the following: priority protection = 27,807 ha; strict control = 20,297 ha; and security utilization = 714 ha (**Figures 8D–F**). As shown in **Figure 8G**, the differences between the two are reflected mainly in that most of the security utilization class is transformed into the priority protection class. Approximately 26.8% of the strict control classes are converted to priority protection classes. However, a small part (approximately 753 ha) of the security utilization class is transformed into the strict control class, potentially because of stricter HT-As vs. RIV-As. According to the data in **Table 4**, the ST–HT evaluation system can not only increase the accuracy rate but also increase the area of the priority protection class paddy field to reduce the economic and restoration pressure caused by overprotection.

These findings suggest that rice plants in karst regions may possess inherent physiological adaptations to high metal backgrounds, potentially involving enhanced root sequestration or altered metal transporter expression, which can be further exploited through biotechnological approaches.

## 4 Discussion

### 4.1 Overarching finding: decoupling of total metal and rice accumulation

Our study reveals critical decoupling between the elevated total concentrations of Cd and As in karst paddy soils of Yunnan and their relatively moderate accumulation in rice grains. Our correlation analysis in **Figure 4** and the variable importance in **Figure 5** quantitatively demonstrate that pH and Fe/Mn oxides are the key drivers of this decoupling for Cd. In this region, total soil As is a primary predictor, unlike Cd which is more influenced by soil chemistry. Specifically, the content of Fe/Mn oxides in soil exerts a significant effect on it. This phenomenon, in contrast to the strong linear relationships often observed in areas contaminated by anthropogenic activities (Williams et al., 2009; Zhao et al., 2009a), underscores the unique biogeochemistry of high geological background areas. The key to reconciling this paradox lies not in the total metal pool but in the innate capacity of the soil to immobilize these metals, which is governed primarily by its alkaline pH and abundant Fe/Mn oxides.

Beyond the geochemical perspective, the observed disparity between high soil metal loads and moderate grain accumulation suggests a potential physiological adaptation of the rice cultivars in this region to metal stress. Understanding such adaptations is necessary for developing targeted strategies to increase plant resilience. While biotechnological tools such as phytohormones or beneficial microorganisms offer promising avenues for mitigating metal toxicity, their application must be guided by a robust understanding of site-specific metal bioavailability and its interaction with plant physiology. This study is therefore aimed at integrating insights from field-based soil–plant transfer models with the principles of plant stress physiology. By deriving thresholds that reflect both the immobilizing capacity of the soil and the response of the rice crop, we can not only prevent unnecessary remediation but also inform and optimize the application of future biotechnological interventions for sustainable rice production in metal-stressed environments.

### 4.2 Immobilization mechanisms: roles of pH and Fe/Mn oxides

The negative correlation between Rice–Cd and pH/F–Fe (**Figure 4**) can be attributed to enhanced specific adsorption and surface complexation of Cd^2+^ on Fe oxide surfaces under alkaline conditions, which carry more negative charges (Bradl, 2004; Antelo et al., 2005). These findings align with those of Li et al (Li et al., 2020), who reported that iron oxides hinder Cd uptake by rice. Conversely, the positive correlation between rice–As and pH appears counterintuitive but is well documented (Smith et al., 1998; Mandal and Suzuki, 2002). Alkaline pH increases the negative charge on soil colloids, promoting the desorption of arsenate (As(V)) anions and thereby increasing As bioavailability. However, the overwhelming dominance of total As (S–T–As) in predicting rice–As (**Figure 5**) suggests that the large source pool in this region is the primary driver, potentially masking the subtle effects of pH on solubility.

The significant role of Mn oxides, particularly their complex form (C–Mn), warrants attention. This positive correlation with rice–CD, although seemingly contradictory, may be indirect. The geochemical properties of Fe and Mn during weathering in this Mn ore-adjacent area lead to the formation of composite oxides with high specific surface areas (Zhou et al., 2015), providing many sorption sites for metals. While Mn oxides themselves may not directly sequester Cd as effectively as Fe oxides do, their presence is a robust indicator of the overall immense metal retention capacity of these soils. The positive correlation between C-Mn and rice-Cd may reflect their common geogenic source or co-occurrence in composite oxides, rather than a direct causal relationship. This correlation warrants further investigation.

### 4.3 Rationalizing the new thresholds: a scientific and practical necessity

The derived ST and HT values, which significantly differ from those of the national RSV/RIV, are not arbitrary but are direct mathematical consequences of the soil properties intrinsic to this karst region of Yunnan. Our ST–Cd concentration (4.54–7.12 mg·kg^-1^) is considerably higher than the standard, which is consistent with the principles of site-specific risk assessment (Ferguson, 1999; Carlon, 2007). This phenomenon is rationally explained by the high total content yet low bioavailability paradigm enforced by high pH and Fe/Mn oxides. By considering only pH, the national standard fails to account for this powerful immobilizing effect, leading to the excessive false positive rates (52.0–66.4%) we observed.

For As, the slightly lower HT–As than RIV–As reflects the increased bioavailability under alkaline conditions, a risk that our model successfully captures. The minimal difference between ST–As and HT–As further underscores that total As content is the paramount factor, a finding that echoes the site-specific nature of soil thresholds (Directive, 2003; Ding et al., 2018). The stark differences between our thresholds and those derived for other crops such as bananas further emphasize the necessity of crop-specific assessments (Pan et al., 2021).

### 4.4 Implications and broader perspectives

The decoupling between high soil metal content and relatively low grain accumulation observed in this study suggests that local rice varieties may have evolved physiological mechanisms to address metal stress, such as increased expression of metal chelators or compartmentalization in vacuoles (Liu et al., 2024b), alternatively, under heavy metal stress, plants experiencing heavy metal stress could avoid absorbing more heavy metal stress by inhibiting root biomass (Wang et al., 2023). These innate tolerance traits provide a genetic resource for developing metal-resistant cultivars through marker-assisted selection or genetic engineering. For instance, overexpression of OsHMA3 or OsNRAMP5 mutants can further reduce Cd accumulation in grains (Zhang et al., 2020; Chang et al., 2022; Wang et al., 2023). Future research could further analyze the biomarkers, such as the key microbial communities in the root system (Cheng et al., 2023), in order to clarify the physiological mechanisms underlying the tolerance of local rice varieties to metals. Our region-specific thresholds, therefore, not only serve as a practical tool for soil management but also identify environments where such biotechnological interventions may be most effective.

The practical implications of implementing the ST-HT system are transformative: a vast area of farmland can be reclassified from the “security utilization (Agricultural land may have the risk of soil pollution of edible agricultural products that do not meet the quality and safety standards. In principle, agrologic control, alternative planting and other safe use measures should be taken)” or “strict control (The risk of soil pollution in agricultural land is high, and it is difficult to reduce the risk of edible agricultural products not meeting the quality and safety standards through safe use measures. In principle, strict control measures such as banning the planting of edible agricultural products and returning farmland to forest should be taken)” category to the “priority protection (The pollution risk of agricultural land is low, and there is no need to take too many safety use measures)” category (**Figure 8G**), thereby liberating considerable economic and administrative resources previously earmarked for unnecessary remediation. At the same time, the agricultural and rural departments could use this threshold as a guidance for drawing the type map of soil cleanliness and apply it to agricultural production guidance. However, it must be acknowledged that the ST-HT system is region-specific, but the method can be extrapolated to other typical karst areas for validation and calibration to further evaluate the broad applicability of the framework. At the national level, based on the macro management and control, formulating unified standards is the internal requirement to facilitate unified management. However, region-specific research is also necessary, which can provide more accurate land management strategies and policy support for regions. This precision land management strategy is both ecologically and economically sustainable.

Our study moves beyond merely deriving new numbers. We provide a replicable framework that integrates field surveys, multivariate regression, and SSD for developing regional criteria elsewhere (For example, typical karst areas such as Guangxi and Guizhou). Future studies should expand this model to include other toxic elements (e.g., Pb and Hg) or staple crops in karst regions. We advocate for a paradigm shift in environmental policy from rigid, national one-size-fits-all standards toward flexible, regionally adaptive thresholds that incorporate key local soil properties. This strategy is not a lowering of safety guards but instead a smart allocation of resources to focus on genuine rather than perceived risks.

## 5 Conclusions

By linking soil geochemistry to plant uptake response, we successfully established and validated a set of regionally tailored soil thresholds for Cd and As in alkaline paddy soils within karst areas of Yunan with high geochemical backgrounds, directly addressing the critical issue of misclassification by the national ‘one-size-fits-all’ standards. Our field survey first revealed the severe inadequacy of the national RSV/RIV system, revealing unacceptably high false-positive rates of 52.0–66.4%, which would trigger unnecessary remediation. We determined that the paradox of high soil metal loads but moderate rice accumulation was governed by the synergistic effect of elevated soil pH and abundant iron/manganese (Fe/Mn) oxides. To overcome this paradox, we integrated multiple linear regression with species sensitivity distribution modeling to derive scientifically defensible safety thresholds (STs) and hazard thresholds (HTs). The newly established thresholds (ST–Cd concentration: 4.54–7.12 mg·kg^-1^; HT–Cd concentration: 9.66–10.12 mg·kg^-1^; ST–As concentration: 91.43–92.30 mg·kg^-1^; and HT–As concentration: 96.90–97.15 mg·kg^-1^) were dramatically more accurate, with the assessment accuracy increasing from 28.1–95.6% to 71.8–100%. This study provides a physiologically informed and regionally adapted framework for establishing Cd and As thresholds. The practical implementation led to a transformative reclassification of farmland, enabling a precise and sustainable risk management strategy that safeguards food security and optimizes resource allocation in geochemically unique regions.

## Data Availability

The raw data supporting the conclusions of this article will be made available by the authors, without undue reservation.
