# Supplementary material for: Integrating soil bioavailability and plant physiological responses to establish region-specific safety thresholds for cadmium and arsenic in rice cultivated in karst regions
Source: Front Plant Sci. 2025 Dec 16;16:1703651. doi: 10.3389/fpls.2025.1703651 (PMC12750342; doi:10.3389/fpls.2025.1703651)
Supplement: Supplementary file 1 [file DataSheet1.docx]

Supplementary Material

# Supplementary Figures


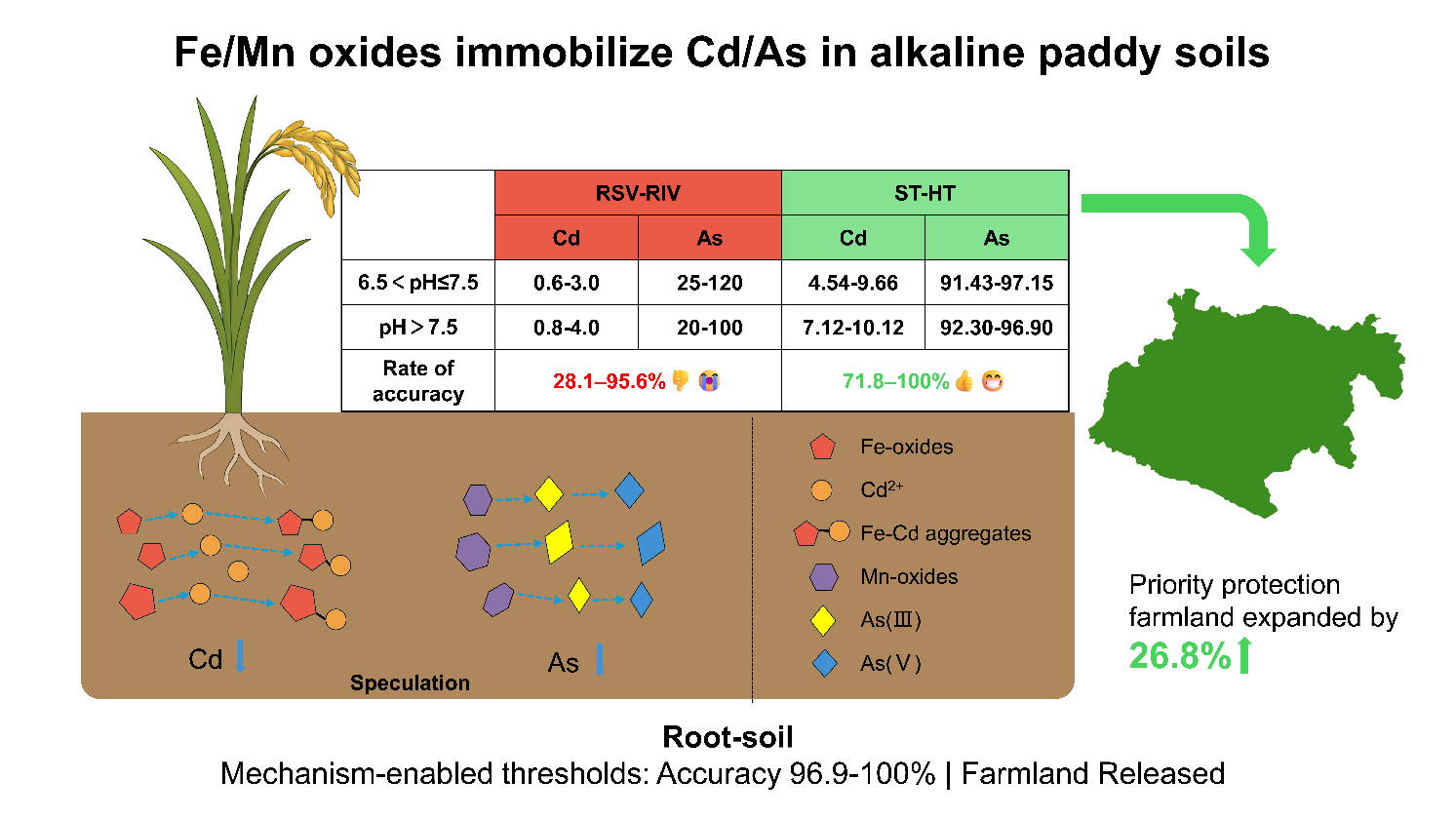


Figure S1. Graphical Abstract

# Supplementary Tables

Table S1. Basic properties of soil and contents of Cd and As in rice

|  | Unit | Min | Max | Arithmetic mean±SD | C.V.(%) |
| --- | --- | --- | --- | --- | --- |
| pH | - | 6.56 | 8.25 | 7.65±0.29 | 3.76 |
| SOM | g⋅kg^-1^ | 9.53 | 70.30 | 34.68±10.53 | 30.36 |
| Soil-Total-Cd（S-T-Cd） | mg⋅kg^-1^ | 0.37 | 15.60 | 2.16±2.51 | 116.40 |
| Soil-Total-As（S-T-As） | mg⋅kg^-1^ | 7.80 | 1,043.22 | 60.18±109.15 | 181.38 |
| Available-Cd（S-A-Cd） | mg⋅kg^-1^ | 0.09 | 3.92 | 0.74±0.80 | 106.92 |
| Available-As（S-A-As） | mg⋅kg^-1^ | 1.50 | 88.61 | 10.66±13.20 | 123.90 |
| Complex-Fe（C-Fe） | mg⋅kg^-1^ | 127.80 | 1,234.40 | 392.07±187.85 | 47.91 |
| Complex-Mn（C-Mn） | mg⋅kg^-1^ | 25.80 | 291.40 | 81.00±41.58 | 51.34 |
| Free-Fe（F-Fe） | mg⋅kg^-1^ | 10,439.00 | 76,247.60 | 34,454.76±13,272.40 | 38.52 |
| Free-Mn（F-Mn） | mg⋅kg^-1^ | 308.10 | 5,973.98 | 1,077.52±589.99 | 54.75 |
| Amorphous-Fe（A-Fe） | mg⋅kg^-1^ | 1,773.20 | 20,084.35 | 4,185.67±2,541.05 | 60.71 |
| Amorphous-Mn（A-Mn） | mg⋅kg^-1^ | 172.22 | 4,019.52 | 975.74±512.30 | 52.50 |
| Rice-Cd | mg⋅kg^-1^ | 0.02 | 0.80 | 0.11±0.13 | 124.44 |
| Rice-As | mg⋅kg^-1^ | 0.04 | 5.68 | 0.33±0.59 | 179.91 |

# Supplementary Methods

Determination of iron-manganese oxides was performed using the Shidan Bao method ((Bao, 2000).

1. Complex-Fe and complex-Mn: Accurately weigh 2 g of soil sample (sifted through a 60-mesh sieve, accurate to 0.01 g) into a 50 mL centrifuge tube. Add 40 mL of 0.1 mol·L^-1^ Na_4_P_2_O_7_(pH≈8.5). Shake at 25°C for 2 hours. centrifuge at 3000 r·min⁻¹ for 6 min, filter the supernatant into a dry plastic vial, and determine the iron-manganese content in the test solution using flame atomic absorption spectrophotometry.
2. Amorphous-Fe and Amorphous-Mn: Accurately weigh 1.5 g (to 0.01 g) of soil sample passed through a 60-mesh sieve into a 100 mL vial. Enclose the vial externally in an opaque bag. Add 75 mL of 0.2 mol·L⁻¹ ammonium oxalate buffer solution (pH≈3.2). securely tie the bag (ensuring the sample remains light-protected to prevent photochemical reactions and maintain sample stability), and shake at 25°C for 4 hours. Immediately decant the sample into a centrifuge tube, then centrifuge at 3000 rpm for 6 minutes. Filter the supernatant into a dry conical flask and seal it tightly. The experimental procedure must be performed continuously. Determine the iron and manganese content in the extract using a flame atomic absorption spectrophotometer.
3. Free-Fe and Free-Mn: Accurately weigh 0.5 g (to 0.0001 g precision) of soil sample (passed through a 60-mesh sieve) into a 50 mL centrifuge tube. Sequentially add 20 mL of 0.3 mol·L^-1^ C_6_H_5_Na_3_O_7_ and 2.50 mL of 1 mol·L^-1^ NaHCO_3_. Incubate at 80°C for 5 min. Add approximately 0.50 g Na₂S₂O₄ and continue incubation for 15 min with constant agitation. After cooling, centrifuge at 3000 rpm for 6 min. Transfer the supernatant to a 100 mL volumetric flask. Repeat this process 1-2 times until the residue appears light gray or grayish-white. Finally, wash the residue 1-2 times with 1 mol·L^-1^ Nacl. Centrifuge the washings and combine with the solution in the volumetric flask. Make up to volume, filter into a dry small white vial, and determine the iron and manganese content in the extract using flame atomic absorption spectrophotometry.

# Reference

Bao S. (2000). *Agrochemical analysis of soil[Chinese]*. China Agriculture Press.
